# An open-access fire detection image dataset for research in computer vision and safety monitoring

**DOI:** 10.1016/j.dib.2026.112801

**Published:** 2026-04-24

**Authors:** Md. Mafiul Hasan Matin, Snigdha Saha, Hridoy Sutradhar, Md Anwarul Islam

**Affiliations:** Department of Computer Science and Engineering, Netrokona University, Netrokona 2400, Bangladesh

**Keywords:** Fire detection, Smoke detection, Computer vision, Deep learning, Image dataset

## Abstract

This article presents the Fire Recognition Image Dataset, a comprehensive collection of images designed to support research in fire detection, smoke recognition, and safety monitoring using computer vision techniques. The dataset was created by systematically selecting publicly available YouTube videos depicting both fire and non-fire scenarios. Sources included uncontrolled fires such as forest, industrial, and household incidents, as well as controlled fire scenes such as campfires, stoves, and candle flames. Frames were manually extracted at regular intervals using Python’s OpenCV library, with redundant, low-quality, or irrelevant frames removed to ensure high-quality data. The dataset comprises 1112 original RGB images categorized into four classes: Real Fire, Smoke, Safe Fire, and Artificial Fire, covering a wide range of environmental conditions, fire types, and perspectives. To increase variability and improve robustness for machine learning applications, a structured data augmentation pipeline was applied using the Albumentations library, including horizontal and vertical flips, rotations, affine shear transformations, brightness and contrast adjustments, and Gaussian noise addition. This augmented the dataset to 6672 additional images, resulting in a total of 7784 images. All images are standardized to 400 × 400 pixels and stored in PNG format. The dataset is organized into class-specific folders with separate subfolders for original and augmented images, enabling straightforward integration into training pipelines. Supporting scripts for frame extraction, preprocessing, and augmentation are provided to ensure reproducibility. This openly accessible dataset offers a valuable resource for researchers developing and benchmarking deep learning models for binary and multiclass fire detection tasks. It can be reused for applications in environmental monitoring, disaster management, and intelligent surveillance, and serves as a benchmark for evaluating computer vision algorithms under diverse real-world and controlled fire conditions.

Specifications Table

**Table:** 

Subject	Computer Sciences
Specific subject area	Deep Learning, Computer Vision, Image Classification
Type of data	Image (.png)
Data collection	Images were extracted from publicly available YouTube videos showcasing a variety of scenarios, including real fires, smoke, safe (no-fire) scenes, and artificial fire simulations. This approach ensured a diverse representation of both real-world and controlled fire situations.
Data source location	Institution: Publicly available YouTube videos Zone: Not applicable Country: Not applicable Latitude and Longitude: Not applicable
Data accessibility	Repository name: Mendeley Data Data identification number: 10.17632/7jk6xh7h6w.4 Direct URL to data: https://data.mendeley.com/datasets/7jk6xh7h6w/4 License: CC BY 4.0
Related research article	None

## 1 Value of the Data

• The dataset provides a comprehensive and diverse collection of real fire, smoke, artificial fire, and safe fire images, enabling researchers to develop and benchmark deep learning models for reliable fire detection and safety monitoring.
• It supports a wide range of computer vision tasks, including binary and multiclass classification, object detection, and image segmentation, making it a versatile resource for both academic and applied research.
• Since the images were sourced from publicly available YouTube videos depicting real-world and controlled fire scenarios, the dataset ensures authentic visual diversity, enhancing model robustness under varied environmental and lighting conditions.
• The augmentation-enhanced dataset allows for experimentation with data augmentation, feature extraction, and model generalization studies, supporting reproducibility and fair comparison between algorithms.
• Being open-access and well-organized, the dataset promotes transparency, collaboration, and reusability, allowing integration with other datasets for research on environmental monitoring, disaster management, and intelligent surveillance systems.
• Beyond the computer vision research community, this dataset can benefit fire safety engineers, emergency response agencies, industrial safety officers, and smart city practitioners by enabling the development of early warning systems for fire outbreaks. It can support real-time surveillance-based fire detection in residential buildings, factories, warehouses, forests, and public infrastructure. Policymakers and disaster management authorities may also leverage models trained on this dataset to improve risk assessment, emergency preparedness, and response strategies, ultimately contributing to reduced fire-related losses and enhanced public safety.

## 2 Background

The rapid and reliable detection of fire and smoke is crucial for reducing property damage, protecting human lives, and supporting environmental monitoring. Manual surveillance methods are labor-intensive and limited in scope, highlighting the need for automated detection systems based on computer vision and deep learning [1].

Several datasets (Table 1) have been developed to advance this research area. The DFS [1] and FASDD [2] datasets provide large-scale annotated fire and smoke imagery, while FIgLib and SmokeyNet [3] contribute real-time wildland fire sequences. For aerial and satellite-based applications, datasets such as FLAME [4], Active Fire Landsat-8 [5], and ONFIRE [9] focus on large-scale fire detection. Others, including HQFSD [6], FSSD [7], and the Flame and Smoke Semantic Dataset [8], offer annotated indoor and outdoor imagery.

**Table 1:** Comparison of major publicly available fire and smoke datasets with the proposed dataset, highlighting differences in scale, data sources, and coverage.

Dataset	Scale (Images / Data Size)	Source Type	Key Focus	Unique Features
FASDD	>100,000 images	Ground + aerial videos	Flame and smoke detection	Large-scale dataset with diverse fire environments
FLAME	Thousands of aerial images	Drone aerial footage	Wildland and controlled burns	High-altitude, drone-based fire surveillance
HQFSD	High-quality indoor/outdoor scenes	Ground cameras	Fire & smoke segmentation	Pixel-level annotated semantic segmentation dataset
ONFIRE	Monthly gridded burned-area maps	Satellite imagery	Environmental monitoring	Satellite-based large-region burned-area mapping
Our Dataset	7784 images	YouTube video frames	Real fire, smoke, safe fire, artificial fire	Real-world everyday scenes, balanced four classes, standardized resolution, strong augmentation pipeline

Despite these contributions, publicly accessible image datasets containing real fire, smoke, artificial fire, and safe fire scenes from everyday environments remain limited [[9], [10], [11], [12]]. To address this gap, the present dataset compiles images extracted from publicly available YouTube videos, offering a diverse and standardized resource for developing and benchmarking deep learning models in fire and smoke detection research.

To highlight the contribution of the proposed dataset, a comparison with existing fire-related image datasets is presented. Unlike datasets such as FireNet and BoWFire, which primarily focus on fire vs. non-fire classification, the proposed dataset introduces a multi-class structure including Real Fire, Smoke, Safe Fire, and Artificial Fire. Additionally, the dataset is constructed from real-world video sources and includes both natural and controlled fire scenarios.

Furthermore, the dataset provides predefined splits, metadata, and augmentation scripts, supporting reproducibility and benchmarking in deep learning research. This combination of multi-class diversity, real-world variability, and reproducibility resources distinguishes the dataset from existing benchmarks.

## 3 Data description

### 3.1 Video/Image selection criteria

The videos and images included in this dataset were selected through a systematic filtering process to ensure relevance, clarity, and labeling accuracy. First, YouTube videos were screened for content containing visible real fire, smoke, artificial fire, and safe fire scenes, while avoiding videos with human subjects, sensitive information, or copyright-restricted material. Video frames were then extracted at uniform intervals, and each frame was visually inspected. Images were accepted if the target phenomenon (fire or smoke) was clearly visible and distinguishable from the background, and rejected if they were blurry, heavily occluded, or lacked sufficient context. Categories such as artificial fire were carefully reviewed to minimize confusion with real fire. Duplicates, low-resolution frames, and frames with unstable lighting or motion blur were also discarded. This filtering process resulted in a balanced four-class dataset with 1112 original images, which were subsequently augmented to create 6672 additional samples. By applying these criteria, the dataset ensures high-quality, accurately labeled images suitable for training and benchmarking fire and smoke recognition models. Since the dataset was constructed using frames extracted from publicly available YouTube videos, certain source-related biases may be present. These include potential overrepresentation of specific environments (e.g., urban or outdoor scenes), recurring camera viewpoints, and lighting conditions commonly found in online video content. Users of this dataset are advised to consider these factors when training and evaluating fire detection models. Future extensions of the dataset may include additional sources and environments to further reduce such biases.

The Fire Recognition Image Dataset is organized into two main folders: “Original_Images.zip” and “Augmented_Images.zip.” Each folder contains four subfolders representing the image classes: *Real Fire, Smoke, Safe Fire*, and *Artificial Fire*. In addition to the four primary classes (Real Fire, Smoke, Safe Fire, and Artificial Fire), a higher-level binary categorization is also defined. Specifically, the classes Real Fire and Smoke are grouped under the “Fire” category, while Safe Fire and Artificial Fire are grouped under the “No Fire” category. Based on this grouping, the dataset is further organized into two super-categories (Fire and No Fire) to support both multi-class and binary classification tasks. The dataset is designed to support research and model development for automatic fire and smoke detection in visual scenes.

All images were collected from publicly available YouTube videos, depicting diverse real-world and controlled fire scenarios. The final dataset includes 1112 original RGB images, which were enhanced through a systematic augmentation pipeline to improve robustness and model generalization. Augmentation techniques included horizontal and vertical flips, brightness and contrast adjustments, random rotations, affine shear transformations, and Gaussian noise addition. This process generated 6672 augmented samples, resulting in a total of 7784 images across all classes. Each image was standardized and resized to 400 × 400 pixels to maintain uniformity in input dimensions.

The dataset follows a simple and interpretable directory structure where each folder corresponds to a single class, removing the need for additional annotation files. All images are stored in PNG format to preserve visual quality without compression artifacts. The dataset contains only environmental scenes and includes no personally identifiable information.

**Fig. 1:**
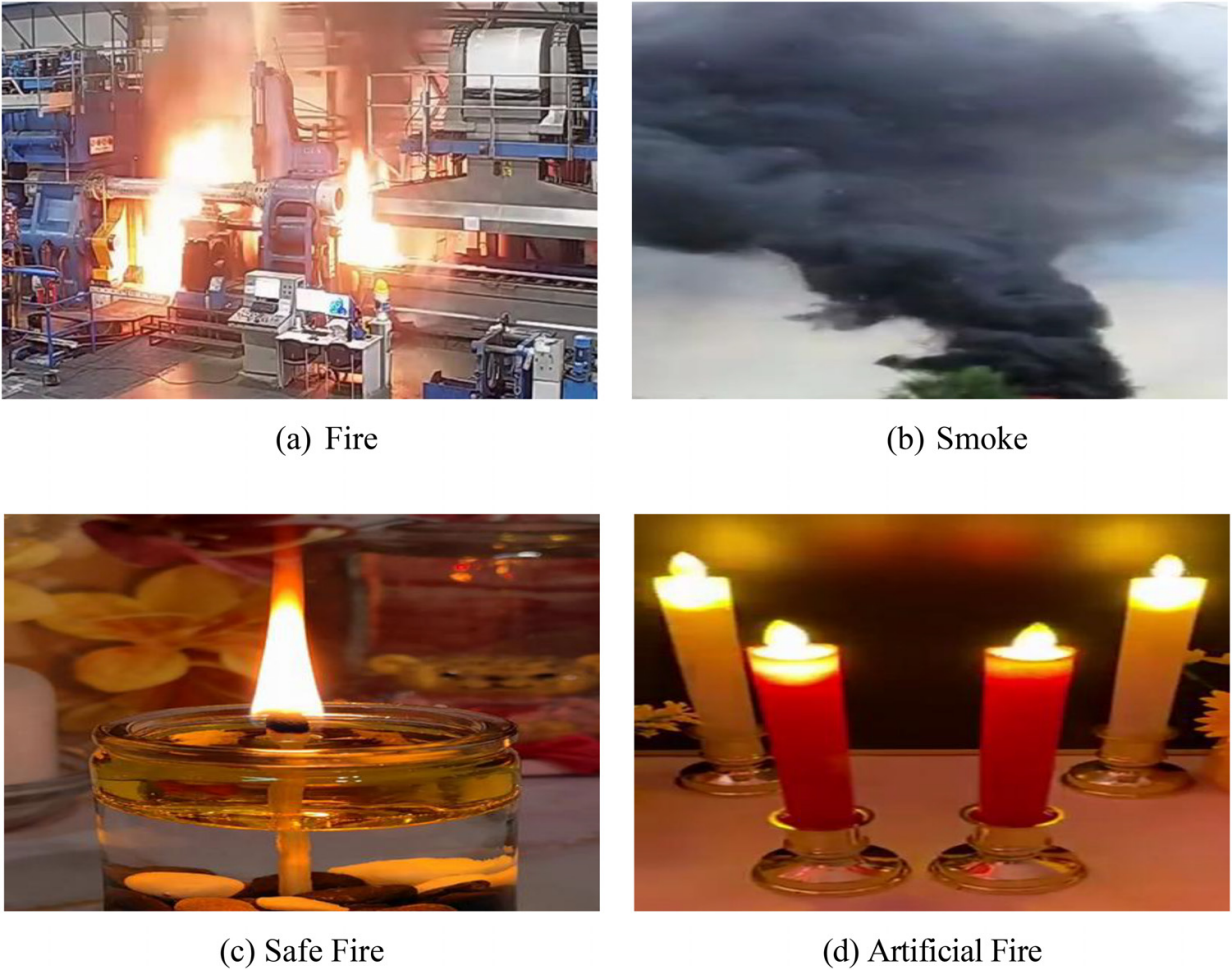
The four categories of image samples included in the *Fire Recognition Image Dataset*. Top row: (a) Fire – images showing active flames; (b) Smoke – images containing visible smoke plumes. Bottom row: (c) Safe Fire – non-hazardous scenes such as candles or stoves; (d) Artificial Fire – simulated or decorative fire displays.

**Fig. 2:**
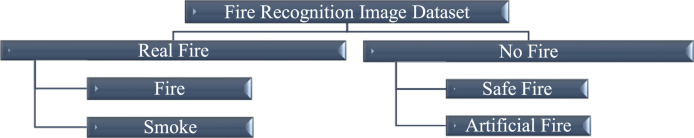
Dataset folder structure.

Although minor variations exist in the number of images across the four classes, the dataset maintains a largely balanced distribution. Table 2 presents the detailed class-wise distribution of both original and augmented images, demonstrating balanced representation across all four categories. This balance helps reduce bias during model training and ensures fair evaluation across real fire, smoke, safe fire, and artificial fire categories. Any remaining imbalance can be easily handled by class weighting or resampling techniques in downstream machine learning experiments. Fig. 1 provides representative samples from each category to visually illustrate the diversity of scenes included in the dataset. Fig. 2 illustrates the hierarchical folder structure used to organize the dataset for efficient access and compatibility with machine learning workflows. For benchmark experiments, we recommend standard dataset splitting strategies such as 70% training, 15% validation, and 15% testing, or alternatively 80% training, 10% validation, and 10% testing. Official train, validation, and test split files for the original images are included in the repository as `train.txt`, `val.txt`, and `test.txt`, corresponding to a 70% / 15% / 15% split to support reproducible benchmarking. These splits are provided as general guidelines to support reproducible evaluation and are not mandatory for dataset usage.

**Table 2:** Statistics of the fire recognition image dataset.

Sl.	Class	Sub-class	Original Images	Augmented Images
1	Fire	Real Fire	283	1698
2		Smoke	273	1638
3	No Fire	Safe Fire	270	1620
4		Artificial Fire	286	1716
Total		**4 categories**	**1112**	**6672**

To provide a clearer overview of the dataset’s diversity, a brief quantitative analysis was conducted on the collected images. Approximately 62% of the images were captured in outdoor environments (e.g., forest fires, street incidents, open-area smoke), while the remaining 38% represent indoor settings such as kitchens, rooms, workshops, and controlled artificial fire scenes. Lighting conditions show notable variation: around 54% of the images fall under daylight or well-lit environments, 33% under low-light or evening conditions, and the remaining 13% contain mixed or unstable lighting (e.g., fluctuating illumination due to flames or smoke). A brightness analysis conducted using the average pixel intensity (0–255 scale) indicates that the dataset covers a broad range of illumination levels, with values spanning from 72 to 198, and an overall mean brightness of approximately 134, reflecting moderate and balanced exposure. These distributions demonstrate that the dataset captures a wide variety of real-world visual conditions, enhancing its suitability for developing robust fire and smoke recognition models.

As the dataset is derived from publicly available YouTube videos, it may contain inherent biases related to content availability and recording preferences. Certain fire scenarios, such as large visible flames or dramatic smoke events, are more likely to be recorded and shared, potentially leading to over-representation of specific visual patterns.

Additionally, variations in environmental conditions such as indoor vs. outdoor scenes, lighting conditions, and camera quality may introduce distributional bias. Although efforts were made to include diverse video sources, the dataset may not fully represent all real-world fire and non-fire scenarios.

Class distribution was controlled during dataset construction; however, some subtle variations within classes may still be underrepresented. These factors should be considered when training and evaluating machine learning models using this dataset.

A README file containing dataset structure and example Python code for loading and usage is provided in the Mendeley Data repository. To support long-term data integrity verification, a manifest file or file-level checksums may be provided in future dataset updates. A metadata file (metadata.csv) describing filename, class label, source video ID, and original/augmented indicator is included in the repository to support reproducibility. The dataset is released under the Creative Commons CC-BY 4.0 license.

Each image in the dataset is linked to its original video source through a structured metadata file (Metadata-2.docx). The metadata includes the source video URL associated with each extracted frame. This mapping ensures traceability and enables researchers to verify the origin of each image, as well as to perform further analysis using the corresponding original video data.

## 4 Experimental Design, Materials and Methods

**Fig. 3:**
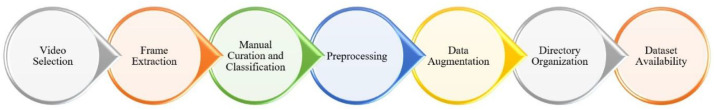
Workflow illustrating the step-by-step process for generating the Fire Recognition Image Dataset, from video selection to dataset organization and augmentation.

The Fire Recognition Image Dataset was created through a structured workflow designed to ensure diversity, quality, and reproducibility. A total of 120 publicly available YouTube videos were systematically selected from multiple categories representing both fire and non-fire scenarios. These included uncontrolled fires, such as forest fires, industrial accidents, and household fires, as well as controlled fire sources, including campfires, gas stoves, and candle flames. Videos were selected based on relevance, clarity, and visual diversity to capture varying environmental conditions, viewpoints, and fire types. Fig. 3 illustrates the complete workflow for generating the Fire Recognition Image Dataset, from initial video selection to final dataset organization and augmentation.

Video frames were extracted using OpenCV at a fixed temporal interval of one frame every 2 s to reduce redundancy while preserving meaningful visual variation. This sampling strategy ensured consistent frame selection across all source videos. Multiple frames may originate from the same source video; however, the fixed extraction interval of 2 s was used to reduce temporal correlation between consecutive frames and to increase scene diversity. Frames were extracted from the selected videos using Python (OpenCV 4.10.0) at regular intervals, balancing dataset size and scene variability. Extracted frames were manually reviewed to remove duplicates, irrelevant content, blurred images, or frames with excessive noise. The remaining images were categorized into four distinct classes Real Fire, Smoke, Safe Fire, and Artificial Fire based on their visual characteristics. Images that did not accurately represent their intended class were discarded to ensure correct labeling and consistency.

**Fig. 4:**
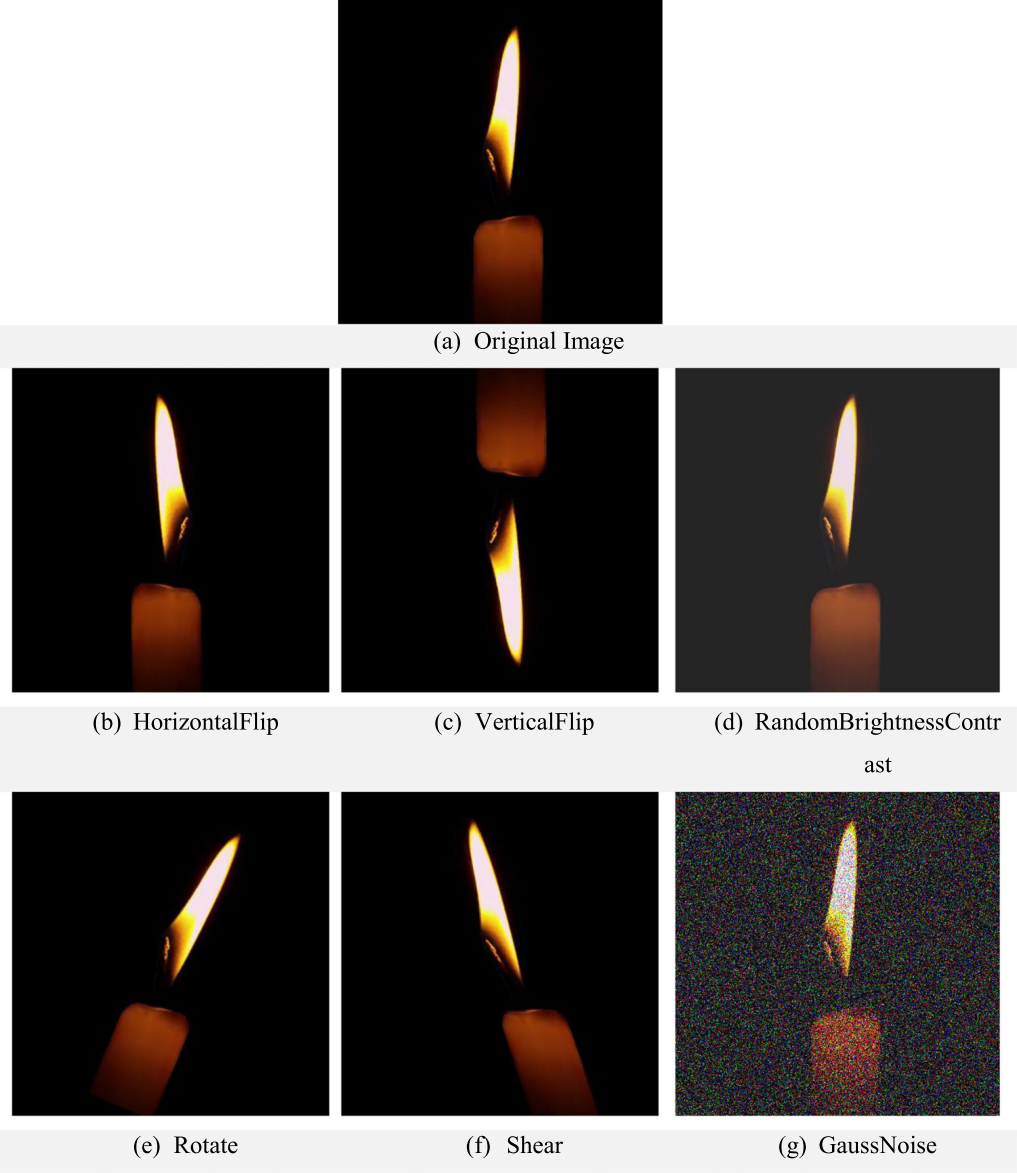
Examples of augmented images illustrating the different transformations applied to the original dataset: (a) original image, (b) horizontal flip, (c) vertical flip, (d) random brightness and contrast adjustment, (e) rotation, (f) shear transformation, and (g) Gaussian noise addition.

All images were standardized to RGB color format and resized to 400 × 400 pixels using OpenCV's INTER_LINEAR interpolation to preserve visual quality. The images were saved in PNG format to preserve visual quality and avoid compression artifacts. To increase dataset diversity and improve model robustness, a data augmentation pipeline was applied using the Albumentations 1.4.8 library. Augmentation techniques included horizontal and vertical flips, random rotations (±30°), affine shear transformations (−20° to +20°), brightness and contrast adjustments, and Gaussian noise addition. This augmentation expanded the dataset from 1112 original images to 6672 augmented images, yielding a total of 7784 images. Fig. 4 presents examples of augmented images generated from the original dataset.

The dataset is organized into a structured folder hierarchy with separate directories for each class, and subdirectories for original and augmented images.

All steps were executed in a Python 3.10 environment, leveraging Google Colab Pro for GPU-accelerated preprocessing and augmentation shown in Table 3. Supporting scripts for frame extraction, preprocessing, and augmentation are included with the dataset to ensure reproducibility. The complete dataset, including all original and augmented images and associated scripts, is publicly available on the Mendeley Data Repository [13], allowing other researchers to use and extend the dataset for fire detection, smoke recognition, and related computer vision tasks.

**Table 3:** 1Summary of image augmentation techniques and parameter settings used in dataset generation

Augmentation Technique	Description	Parameter Range / Setting	Probability (p)
Horizontal Flip	Flips the image horizontally to simulate mirrored viewpoints	Left–right flip	0.5
Vertical Flip	Flips the image vertically to increase viewpoint variation	Top–bottom flip	0.3
Random Brightness & Contrast	Randomly adjusts image brightness and contrast to simulate different lighting conditions	Automatically selected by Albumentations	0.3
Rotation	Rotates the image to simulate camera angle variation	−30° to +30°	0.5
Shear (Affine Transformation)	Applies shear distortion along X and Y axes to simulate perspective changes	−20° to +20°	0.5
Gaussian Noise	Adds random Gaussian noise to improve robustness against sensor noise	Variance: 10.0–50.0	0.3

The dataset is constructed from publicly available YouTube videos, making it a secondary data source. The authors performed video selection, frame extraction, filtering, annotation, and augmentation to create a structured and research-ready dataset. No copyrighted or restricted content was used beyond publicly accessible materials.

All extracted image frames were manually annotated into four predefined classes: Real Fire, Smoke, Safe Fire, and Artificial Fire. The annotation process was carried out by two independent annotators with prior knowledge of computer vision and fire detection concepts. Each annotator labeled the images independently based on visual characteristics such as flame presence, smoke patterns, and contextual cues.

To ensure labeling quality and consistency, a subset of the dataset was cross-validated by both annotators. In cases of disagreement, the annotations were reviewed jointly and resolved through discussion until a consensus was reached. The overall inter-annotator agreement was approximately 95%, indicating a high level of consistency in the labeling process.

Ambiguous or unclear frames were excluded during the manual filtering stage to maintain dataset reliability and class purity.

## Limitations

The dataset was compiled exclusively from publicly available YouTube videos, which provides practical accessibility but also introduces several inherent limitations. Firstly, the dataset may exhibit bias toward fire types, environments, and camera perspectives that are more commonly uploaded online, potentially underrepresenting industrial fires, rare fire scenarios, or specialized settings. Secondly, video frames vary in resolution, compression, lighting, and color balance, which may reduce the generalizability of models trained solely on these images. Thirdly, although the dataset includes extensive augmentation, its overall size is modest compared to large-scale image datasets, limiting the training of extremely deep neural networks without supplemental data. Fourthly, some categories, such as artificial fire or simulated smoke, can visually resemble real fire, which may introduce subtle labeling ambiguities despite careful manual curation. Furthermore, the dataset predominantly reflects what is visible to consumer-grade cameras, so extreme conditions such as low-light scenarios, high dynamic range lighting, or perspectives captured by specialized sensors (infrared, thermal) are not represented.

In terms of practical deployment, these limitations imply that models trained on this dataset may face challenges in real-time fire detection or in applications with diverse environmental conditions. To mitigate these issues, additional preprocessing steps may be required, including resolution standardization, color normalization, noise reduction, and temporal frame selection for video sequences. Additionally, domain adaptation or fine-tuning with environment-specific images is recommended to improve robustness and generalization for indoor, outdoor, and surveillance-based fire monitoring applications. Despite these constraints, the dataset remains valuable as a benchmark for general-purpose fire and smoke recognition models using consumer-grade cameras in real-world conditions.

## Ethics Statement

In this article, there is no research involving human or animal subjects conducted by the authors. The datasets used in this article are publicly accessible. Proper citation of these datasets is important when using them.

The images included in this dataset were collected through web scraping from publicly available online sources that explicitly allow data access and reuse for non-commercial and research purposes according to their Terms of Service. No data were taken from social media platforms, user-restricted areas, or content requiring login credentials. The collected images represent only open-access visual data of fire and non-fire scenes without any personally identifiable or sensitive information. Each image was manually reviewed to ensure compliance with copyright and data protection standards. The dataset is published solely for academic research and educational use, adhering to fair-use and ethical data-sharing principles.

## CRediT Author Statement

**Md. Mafiul Hasan Matin:** Software, Supervision, Writing – review & editing; **Snigdha Saha:** Conceptualization, Data curation, Methodology; **Hridoy Sutradhar:** Data curation, Validation, Formal analysis, Visualization; **Md Anwarul Islam:** Investigation, Resources, Supervision.

## Funding

This research did not receive any specific grant from funding agencies in the public, commercial, or not-for-profit sectors.

## Data Availability

Mendeley DataFire Recognition Image Dataset (Original data)

## References

[1] Wu S., Zhang X., Liu R., Li B. (2023). A dataset for fire and smoke object detection. Multimed. Tools. Appl..

[2] Wang M., Jiang L., Yue P., Yu D., Tuo T. (2023). FASDD: an open-access 100,000-level flame and smoke detection dataset for deep learning in fire detection. Earth Syst. Sci. Data Discuss..

[3] Dewangan A., Pande Y., Braun H.W., Vernon F., Perez I., Altintas I., Cottrell G.W., Nguyen M.H. (2022). FIgLib & SmokeyNet: dataset and deep learning model for real-time wildland fire smoke detection. Remote Sens..

[4] Shamsoshoara A., Afghah F., Razi A., Zheng L., Fulé P.Z., Blasch E. (2021). Aerial imagery pile burn detection using deep learning: the FLAME dataset. Comput. Netw..

[5] De Almeida Pereira G.H., Fusioka A.M., Nassu B.T., Minetto R. (2021). Active fire detection in Landsat-8 imagery: a large-scale dataset and a deep-learning study. ISPRS. J. Photogramm. Remote Sens..

[6] Qian J., Hong C., Zhang K., Huang J. (2024). Proceedings of the 2nd International Workshop on Multimedia Content Generation and Evaluation: New Methods and Practice.

[7] Hou F., Rui X., Chen Y., Fan X. (2023). Flame and smoke semantic dataset: indoor fire detection with deep semantic segmentation model. Electronics.

[8] Gincheva, A., & Turco, M. (2023). ONFIRE dataset: monthly gridded burned area data. ONFIRE Dataset: Monthly Gridded Burned Area Data.

[9] Wang M., Yue P., Jiang L., Yu D., Tuo T., Li J. (2025). An open flame and smoke detection dataset for deep learning in remote sensing based fire detection. Geo-spat. Inf. Sci..

[10] Han X., Pu N., Feng Z., Bei Y., Zhang Q., Cheng L., Xue L. (2024). Chinese Conference on Pattern Recognition and Computer Vision (PRCV).

[11] Gagliardi A., Saponara S. (2020). Advised: advanced video smoke detection for real-time measurements in antifire indoor and outdoor systems. Energies. (Basel).

[12] Elhanashi A., Essahraui S., Dini P., Saponara S. (2025). Early fire and smoke detection using deep learning: a comprehensive review of models, datasets, and challenges. Appl. Sci..

[13] Saha S., Sutradhar H., Islam M.A., Matin M.M.H. (2026). Fire recognition image dataset. Mendel. Data.

